# Intentional self-harm seen in psychiatric referrals in a tertiary care hospital

**DOI:** 10.4103/0019-5545.43633

**Published:** 2008

**Authors:** Partha Pratim Das, Sandeep Grover, Ajit Avasthi, Subho Chakrabarti, Savita Malhotra, Suresh Kumar

**Affiliations:** Department of Psychiatry, Postgraduate Institute of Medical Education and Research, Chandigarh - 160 012, India

**Keywords:** Depression, insecticide poisoning, intentional self-harm

## Abstract

**Background:**

Intentional self-harm is common, through out the world; however, there is scanty data from India.

**Aims:**

To study the sociodemographic and clinical profile of subjects with “intentional self-harm” referred to consultation-liaison psychiatric services for evaluation in a tertiary care hospital.

**Design:**

Retrospective chart review.

**Materials and Methods:**

For this study, the consultation-liaison register of Department of Psychiatry was screened to obtain data of all patients who were referred to psychiatry referral services and were diagnosed as “intentional self-harm” while they were admitted in Nehru Hospital, Postgraduate Institute of Medical Education and Research (PGIMER), Chandigarh during the period of 2000-2005. The case notes of these patients were reviewed for obtaining the sociodemographic profile and clinical profile for the current study.

**Results:**

Majority of the subjects were married (61%), educated beyond matriculation (75%), were employed or retired (53.6%), belonged to Hindu (87%), nuclear family (64.5%) of middle socioeconomic status (85%) and came from urban background (53%). Most common reasons/precipitating events prior to intentional self-harm were interpersonal problems with family members (39.2%), followed by interpersonal problems with spouse (16.9%). The most common method of intentional self-harm used was consumption of insecticides (44.6%), followed by use of corrosives (17.5%). Half of the sample (48.2%) did not fulfill criteria for any axis-1 or axis-2 psychiatric diagnosis at the time of assessment and most common psychiatric diagnosis was depression (30.7 %).

**Conclusions:**

Nearly half of the subjects who present to a tertiary care hospital with intentional self-harm do not have diagnosable psychiatric illness.

## INTRODUCTION

Various terms like “attempted suicide,” “deliberate self-poisoning,” “deliberate self-injury,” and “parasuicide” have been used interchangeably,[1] to define subjects who present with self-harm. Although some of the authors[2,3] have tried to separate these categories based on the suicidal intent at the time of the act, clinically it is not possible to do so in every case and hence, the most accepted term in recent times to describe such behavior is “Deliberate Self-Harm,” which is defined as “self-poisoning or injury, irrespective of the purpose of the act”[4] and it is one of the top five causes of acute medical admissions for both men and women.[5] As in many countries, deliberate self-harm in India is an unrecognized, hidden, and a silent epidemic. Although the literature is very scant from Indian subcontinent, yet the available data suggest that the number is rising steadily[6,7] and that the risk factors associated and methods employed for suicide attempt/self-harm are strikingly different from those reported in Western data.[8]

The psychosocial milieu of the developing countries is different from that of the developed countries. Indian society is nontolerant to suicide, views it as an act of cowardice and betrayal.[9] Compared to the West, many of Indian patients suffering from various mental illnesses live with their families[10] are married.[11] In India, poverty, illiteracy, and unemployment are much more common and social security system is not existent compared to the West.[8] Another important precipitant for suicide/self-harm in India is dowry disputes, which is not seen in Western culture. Further, compared with the west there is scarcity of mental health services. Indians also rely heavily on religious beliefs, prayers, fasting, and faith in divine to provide solace and maintain hope. The basic tenet of Hindu philosophy is to maintain a fatalistic attitude, and consequently many resign themselves to their current situation rather than striving actively to change it.[6] Due to the same, the findings of developed countries may not hold true for India.

In many countries including India, subjects who harm themselves frequently present to hospital emergency for medical complications arising as a result of self-harm. Hence, they form an important group to understand the psychosocial profile of patients who harm themselves. With this background, aim of our study was to study the sociodemographic and clinical profile of subjects with “intentional self-harm” referred to consultation-liaison psychiatric services for evaluation in a tertiary care hospital.

## MATERIALS AND METHODS

### Setting

The study was carried out at the Postgraduate Institute of Medical Education and Research (PGIMER), Chandigarh, providing services to a major part of North India. PGIMER is a multispecialty tertiary-care teaching hospital; there is extensive cross-referral among the various departments of the Institute for specific problems. Department of Psychiatry of the Institute runs a round-the-clock, three-tier system for providing emergency psychiatry cover for the entire hospital including its Emergency Medical and Surgical outpatients. This system was initially run on written calls. Later, this system was supported by the paging system of the Institute. All the referred cases are initially evaluated by a trainee psychiatrist (junior resident), under the supervision of a qualified psychiatrist (senior resident). The cases are evaluated for the presence of psychiatric morbidity, and if it is present, it is assessed whether it is attributable to a physical illness or not. The diagnoses are made as per the ICD-10.[12] The cases evaluated by the junior and senior residents are reviewed by a consultant psychiatrist and an appropriate treatment plan is formulated and carried out. In addition to documentation of the patient status in the referring department's file, the psychiatry referral team maintains its own separate records for all the patients seen. After discharge from the hospital by the referring unit, the psychiatry referral file is transferred to the Psychiatry OPD record section and the patient is followed up by the same junior resident/senior resident/consultant who had seen the patient in consultation-liaison, to maintain the continuity of care.

In addition to this, information of all the calls received from various wards is also summarized in a referral register under the following headings - age, sex, source of referral, diagnosis of the physical condition, reason for psychiatry referrals, psychiatric diagnosis, management done, and outcome. This register is monitored for completeness weekly by the designated Consultant Incharge of Psychiatry Referral Services.

Whenever a patient is admitted to hospital with intentional self-harm/suspected self-harm of any kind, the psychiatric referral team is consulted for detailed psychiatric evaluation. Besides the documentation of sociodemographic profile, the detailed psychiatric evaluation of such patients involves obtaining a psychiatric history, immediate precipitating event prior to self-harm, method used, family history of psychiatric disorders including suicide or suicide attempts, current mental status examination, risk of future attempt, etc. After the initial evaluation, these patients are subsequently followed up in the inpatient setting till they are physically stable. After this, depending on the mental status examination and risk of future attempt, these patients are either transferred to psychiatry ward or are followed up in psychiatry OPD. The psychiatry management usually involves treatment of axis-1 diagnosis by pharmacotherapy and psychotherapy.

For this study, the referral register was screened to obtain data of all patients who were referred to psychiatry referral services and were diagnosed to have “intentional self-harm” (as per ICD-10[12]) while they were admitted in Nehru Hospital, PGIMER, Chandigarh during the period of 2000-2005. Usually, the diagnosis is entered in the referral register at the time of discharge of the patient, which provides sufficient time to obtain information and conduct multiple mental status examinations and corroboration of history from various informants. The files of patients whose diagnosis included “intentional self-harm” were reviewed for obtaining the sociodemographic profile and clinical profile for the current study.

## RESULTS

During the study period (2000-2005), a total of 3092 cases were referred to psychiatric referral services, out of which 166 cases were of intentional self-harm, which gives a prevalence figure of 5.36% of intentional self-harm cases in psychiatry referrals.

### Sociodemographic profile

There was nearly equal distribution of males (53%) and females (47%) in the sample. Majority of the subjects were married (61%), educated beyond matriculation (75%), were employed or retired (53.6%), belonged to Hindu (87%), nuclear family (64.5%) of middle socioeconomic status (85%) and came from urban background (53%).

The mean age at intentional self-harm of the sample was 29 years (SD - 12.11), with a range of 12-76 years. There was no significant difference between males and females in terms of mean age at intentional self-harm. When the age at intentional self-harm was analyzed in terms of groups, nearly half of subjects (48%) were in the age group 20-30 years and one-fifth (19%) were aged between 10 and 20 years.

### Clinical features

More than three-fourth of the sample (78.3%, *n* = 130) had not consulted any psychiatrist in the past. Among the diagnosed cases, the most common diagnoses were affective disorders (39%). There was history of previous intentional self-harm in 9% of cases. When the subjects were assessed after the intentional self-harm, nearly half (52%) of them were diagnosed to have some psychiatric disorder, with depressive disorder being the most common [Table 1]. About half (48%) of the sample did not fulfill any axis-1 diagnosis and they were labeled as only having “intentional self-harm” as per ICD-10 diagnostic code of X60-84. Most of the subjects (94.6%) did not have any family history of mental illness, but most of them (85.5%) had a family history of substance use.

**Table 1:** Clinical profile of patients seen in psychiatry referrals with intentional self-harm

Variable	*n* (%)
Past history of intentional self-harm	15 (9.0)
Current psychiatric diagnosis	-
Intentional self-harm^a^	80 (48.2)
Depression (unipolar/bipolar)	51 (30.7)
Adjustment disorder	14 (8.4)
Schizophrenia	8 (4.8)
Emotionally unstable personality disorder	7 (4.8)
Other psychiatric diagnosis	6 (3.6)
Reason/precipitating event prior to attempt	
Interpersonal relationship problems with family members	65 (39.2)
Interpersonal relationship problems with spouse	28 (16.9)
Job-related stress	20 (12.1)
Delusions	9 (5.4)
Failure in exams	7 (4.2)
Broken love affair	5 (3.1)
Interpersonal relationship problems with friends	2 (1.2)
Insufferable pain	1 (0.6)
Molestation attempt by a close relative	1 (0.6)
Depressive cognitions	28 (16.9)
Method used	
Insecticides	74 (44.6)
Corrosives	29 (17.5)
Psychotropic	21 (12.7)
Self-immolation	18 (10.8)
Others	24 (14.4)

a Patients were diagnosed “intentional self-harm” only as per ICD-10, when they did not fulfill the criteria for any axis-I or axis-II disorder.

### Clinical features related to intentional self-harm

Majority of the subjects (78%) had a precipitating event prior to the intentional self-harm, most common of which was interpersonal problems with family members other than spouse (38%), followed by interpersonal problems with spouse (16.9%). The most common method of self-harm was consumption of insecticides (44.6%), followed by use of corrosives (17.5%) and use of psychotropic drugs (12.7%). About 17% of patients had harmed themselves after consuming alcohol. Out of the psychotropics, sedative-hypnotic group was the commonly used drug for overdose [Table 1]. Use of insecticides was almost equal in both genders (48% for males and 51% for females). However, more fatal methods like stabbing self with a sharp instrument, guns shot, jumping in front of train were observed only in males (*n* = 8, 4.8%).

Comparisons based on the type of method used showed that majority of subjects who took overdose of psychotropics were suffering from depression whereas subjects who used corrosives and organophosphorous did not have a diagnosable psychiatric illness at presentation. In more than half of the cases (56.6%), intentional self-harm did not lead to any severe medical complications. Sixteen percent of the patients developed delirium, 10% developed postcorrosive esophageal ulceration and 3.6% developed hypoxic ischemic encephalopathy as a complication to the methods used for intentional self-harm.

Eight patients died during the inpatient stay. Mortality was associated with methods like burns (four patients), poisoning (n=2), stab injuries (n=1) and hanging (n=1).

### Comparison statistics

#### Gender differences

On comparison, it was found that female subjects who indulged in self-harm behavior were significantly more educated than males (*t* value= 2.50*, DF = 164; *P* < 0.05). In females, the intentional self-harm was more frequently preceded by depressive cognition (present in 19 females compared to 9 males) and this difference was statistically significant (Chi square value 5.889*, DF = 1; *P* < 0.05). Further, any axis-I or axis-II diagnosis was more frequently seen in females (47 females had a psychiatric diagnosis compared to only 34 males) and the difference was statistically significant (Chi square value 7.73**, DF = 1; *P* < 0.01).

#### Subjects with and without axis-1 and axis-2 diagnosis

There was no statistically significant difference in the sociodemographic profile between those with and without any axis-I or axis-II diagnosis. There was difference in the immediate reason/precipitating event proceeding intentional self-harm. As expected subjects with a psychiatric diagnosis more frequently reported depressive cognition, whereas subjects without any axis-I or axis-II diagnosis more frequently reported interpersonal problems leading to intentional self-harm. Patients with psychiatric diagnosis more frequently used methods like psychotropic overdose (Chi square value 9.95**, DF = 1; *P* < 0.01), whereas subjects without any axis-I or axis-II diagnosis more frequently used insecticide (Chi square value 4.93*, DF = 1; *P* < 0.05).

## DISCUSSION

The present study was a retrospective chart review for the sociodemographic and clinical profile of subjects with intentional self-harm presenting to a tertiary care hospital referred to psychiatry consultation-liaison services for psychiatric evaluation. The sociodemographic profile of our sample was similar to that in other studies from India.[6,13] Majority of our sample comprised of young subjects (mean age 29 years) suggesting that they constitute a vulnerable group. This observation is identical with previous literature from India and the West.[6,14] The male predominance in our sample is also in keeping with previous studies from India.[6,7,15] However, this is at variance with Western literature wherein majority of attempters are females.[14] Predominance of male subjects can be understood to be arising out of sociocultural and occupational roles that Indian males have to perform as discussed by Latha *et al*.[6] Although the role of women has recently changed in some ways over the years, with more females taking up jobs, yet Indian society continues to harbor male dominance. The culturally glorifying position of the males puts them to more stress and expectations. Marriage is an almost universal phenomenon in India and predominance of married subjects in our sample could be explained by the same.[16] Similar results are shared by the multinational study by Fleischmann *et al.*[17] in which subjects from Indian center who attempted suicide/indulged in self-harm were more frequently married than single. The social structure of India is not static, especially if one looks at the recent trends. More people are living in nuclear than in the joint family setups.[16] The nuclear family setups are probably not able to provide as much support and are leading to increase in level of stress with consequent self-harm. This is endorsed by the finding of predominance of subjects from nuclear families in our sample. Predominance of cases from urban middle socioeconomic families also perhaps reflects the transition of the Indian society and the stress associated with it, though, another pragmatic reason for predominance of urban subjects could be accessibility to the hospital leading to greater treatment seeking behavior.

### Intentional self-harm related variables

The commonest method employed to execute self-harm was insecticide poisoning. Similar findings have been reported from elsewhere in India[6,7,18] and other low- and middle-income countries.[17] Unrestricted availability of the insecticide is the probable cause. This implies that there is need to enforce legislative control over the availability of the insecticides. There is also need to carry out parallel campaigns on educating the public in this regard.

Studies from the West[19,20] as well as from India[15,21] have reported recent life events to be important risk factors for suicide attempts/intentional self-harm. Siwach and Gupta[15] reported marital disharmony, economic hardships and scolding/disagreement with other family members as the major precipitating factors. Interpersonal problems and job-related problems found in our study are in line with the same. These results imply that there is a need to establish counseling services with focus on high-risk families. Failure in the examination was an important antecedent in student population; hence, the suicide prevention programs should include skills development for children and families to handle the multitude of issues related to academic pressure.

A significant proportion of our sample did not meet criteria for any axis-I psychiatric diagnosis. Similarly, Parker *et al.*[22] also reported that 45% of subjects who attempt suicide/self-harm themselves do not have a diagnosable psychiatric illness. These findings suggest that intentional self-harm is not only limited to psychiatrically ill subjects, but it is also used by so-called normal persons as a coping mechanism under stress to communicate their needs and distress.

Diagnosis of depressive episode or adjustment disorder in 40% subjects matches the existing literature from India.[22] It is to be noted that 52% of our subjects had diagnosable psychiatric illness, but most of them had not sought treatment for the same. This implies that there is an urgent need to promote education regarding the nature of psychiatric disorders and their treatability across the community to allow their early detection and timely treatment thereby minimizing suicide attempts/intentional self-harm. Stigma reduction programs, effective skills on the part of primary care and family physicians for identification and management of potential suicidal persons, coverage of unreached areas in terms of better accessibility of mental health care should be promoted. Suicide prevention must form an integral part of community-based mental health care activities.

### Limitations

Our study has some limitations. Our sample cannot be considered as truly representative of the population as all cases who present with intentional self-harm are not referred for psychiatric consultations; some are discharged prior to assessment and in some cases families do not disclose the facts to the treating agencies due to legal consequences. Another limitation of the study is the retrospective nature of the same. Hence, the findings should be interpreted in this background. Our study also did not look at the suicidal intent of the subjects at the time of intentional self-harm.

## CONCLUSIONS

Young age group represents the most vulnerable group in need. Half of the patients were diagnosed with psychiatric illness at presentation, which clearly argues for need of early, prompt diagnosis and treatment of such cases so as to preempt such attempts. Depression remains the most common diagnosis; its early identification and proper treatment can lead to reduction in suicide attempts/intentional self-harm and perhaps completed suicides. Our findings also suggest that intentional self-harm is also used by so-called normal persons as a coping mechanism under stress to communicate their needs and distress. Hence, promoting healthy coping mechanism and reduction in stress is required to reduce self-harm. As is evident from the study, modifying the interpersonal relationship problems in the family might help in preventing many of suicide attempts/intentional self-harm. There is also need to develop clear policy for sale and possession of insecticides.
